# Mental Health Admissions to Paediatric Wards Study (MAPS): a protocol for the analysis of Hospital Episode Statistics (HES) data

**DOI:** 10.1136/bmjpo-2023-002352

**Published:** 2024-01-29

**Authors:** Lee Duncan Hudson, Joseph Ward, Adriana Vázquez-Vázquez, Kate Settle, Francesca Cornaglia, Faith Gibson, Kirsty Phillips, Gabrielle Mathews, Helen Roberts, Damian Roland, Dasha E Nicholls, Holly Elphinstone, Russell Viner

**Affiliations:** 1 Population, Policy and Practice Research Programme, UCL Great Ormond Street Institute of Child Health, London, UK; 2 Great Ormond Street Hospital for Children NHS Trust, London, UK; 3 Queen Mary University of London, London, UK; 4 University of Surrey, Guildford, UK; 5 CYP Transformation Team, NHS England and NHS Improvement London, London, UK; 6 SAPPHIRE Group, Population Health Sciences, Leicester University, Leicester, UK; 7 Paediatric Emergency Medicine Leicester Academic (PEMLA) Group, Children's Emergency Department, Leicester Royal Infirmary, Leicester, UK; 8 Division of Psychiatry, Imperial College London, London, UK

**Keywords:** child psychiatry, epidemiology, psychology, statistics, adolescent health

## Abstract

**Introduction:**

Children and young people (CYP) presenting with a mental health (MH) crisis are frequently admitted to general acute paediatric wards as a place of safety. Prior to the pandemic, a survey in England showed that CYP occupied 6% of general paediatric inpatient beds due to an MH crisis, and there have been longstanding concerns about the quality of care to support these patients in this setting. Mental Health Admissions to Paediatric Wards Study aims to generate a theory of change (ToC) model to improve the quality of care for CYP admitted to acute paediatric services after presenting in a MH crisis.

**Methods and analysis:**

We will undertake a national (England), sequential, mixed methods study to inform a ToC framework alongside a stakeholder group consisting of patients, families/carers and healthcare professionals (HCPs). Our study consists of four work packages (WP) undertaken over 30 months. WP1 is limited to using national routine administrative data to identify and characterise trends in MH admissions in acute paediatric wards in England between 2015– 2022.

**Ethics and dissemination:**

WP1 received ethical approval (Ref 23/NW/0192). We will publish the overall synthesis of data and the final ToC to improve care of CYP with MH crisis admitted to general acute paediatric settings. As coproducers of the ToC, we will work with our stakeholder group to ensure wide dissemination of findings. Potential impacts will be on service development, new models of care, training and workforce planning.

**WHAT IS ALREADY KNOWN ON THIS TOPIC**

- There is evidence that both the number of paediatric admissions and the severity of MH crisis in CYP have increased.
- HCPs from children’s wards are reporting that they are finding supporting CYP admitted with MH problems challenging.

**WHAT THIS STUDY HOPES TO ADD**

- This study will provide a comprehensive national analysis describing trends and characteristics of acute admissions due to MH problems in CYP.
- This study will provide the financial burden associated with MH admissions and an analysis of the community MH support and the local burden of admissions.
- This study will generate a ToC model to positively impact on quality of care for CYP who are admitted to paediatric service because of a MH crisis.

**HOW THIS STUDY MIGHT AFFECT RESEARCH, PRACTICE OR POLICY**

- By producing a ToC approach, we expect to generate a system map to identify transformation plans to share with policymakers, commissioners, service leads, and professionals.
- Our data and outputs will enable advocating for and improving cultural views on CYP with MH crises as part of the acute paediatric system.

## Introduction

Children and young people (CYP) presenting with a mental health (MH) crisis are frequently admitted to general acute paediatric wards as a place of safety, despite not always having the resources or training.^1 2^ Before the pandemic, a survey carried out in 2019 with 60% of acute paediatric services in England found that 6% of general paediatric beds were occupied by CYP with MH problems.^3^ Moreover, data from London suggest that the management of CYP with MH problems was one of the main challenges for acute children’s services.^3^

The rise in MH problems among CYP during the COVID-19 pandemic has been also well described.^4 5^ Recent national data report that being at high risk of MH problems rose from 1 in 9 in 2017 to 1 in 6 by 2021, with a doubling of the proportion of CYP at risk of eating problems over that same period.^6^ During the first wave, acute services became ‘default providers’ where community or inpatient Child and Adolescent Mental Health Services (CAMHS) services were not accessible, and during the third wave, admissions to acute wards appeared to peak.^7^ This mismatch of greater distress and reduced access led to increases in already unmet needs.^8^

There is a striking lack of information available to guide care and service delivery for the rapidly growing issue of CYP with MH problems being admitted and managed on acute paediatric wards. Evidence on interventions to avoid inpatient admissions for CYP presenting in MH crisis is poor and limited,^9^ meaning that CYP are likely to continue to need to be admitted in crisis, with paediatric wards a commonplace while awaiting assessment given the lack of direct access to a specialist MH ward form most CYP.

Overall, MH admissions to acute paediatric wards are a long-standing issue that has been identified as a leading safety and quality concern for acute paediatric providers for some years.^1 3^ The Mental Health Admissions to Paediatric Wards Study aims to generate a theory of change (ToC) model to improve the quality of care for CYP admitted to acute paediatric services after presenting in an MH crisis. Our study consists of four work packages. Here, we describe work package 1 (WP1), which is limited to using national routine administrative data to identify and characterise trends in MH admissions in acute paediatric wards in England between 2015 and 2022. WP2 and 3 were described separately.^10^

## Methods and analysis

We will use a ToC approach as our framework, which uses logic (quantitative) data and coproduction (qualitative) data to map change. This approach has been applied to a range of areas of health and social care improvement settings.^11–13^

**Figure 1:**
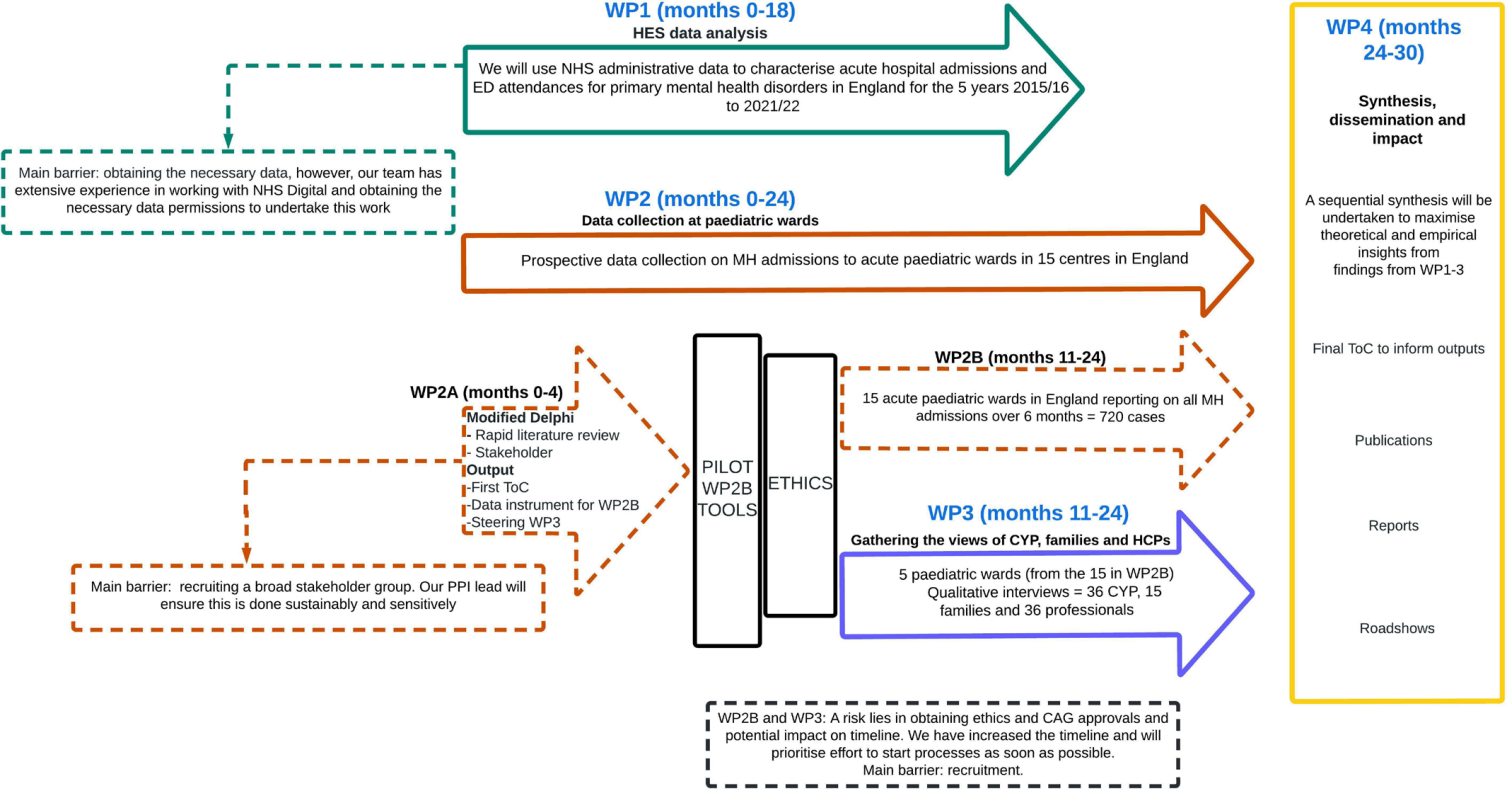
WP1 data flow diagram. CYP, Children and Young People; HES, Hospital Episodes Statistics; MH, mental health; NHS, National Health Service; UCL, University College London.

We will undertake a national, sequential, mixed methods study to inform our ToC framework alongside a stakeholder group consisting of patients, families/carers and HCPs. Three work packages will deliver the types of evidence needed to inform our ToC (figure 1). Here we describe the study design for WP1.

### Objectives

The analysis of routine administrative data will be highly informative regarding national trends and characteristics of CYP admitted. The aims are to:

1. Describe trends in MH admissions to acute inpatient services, attendances to accident & emergency (A&E) and outpatient appointments related to MH within England 2015–2022 (or the latest available data) in CYP aged 5–18.
2. Understand the financial burden of MH admissions to acute paediatric wards among CYP aged 5–18 in England.
3. Examine the role of primary and community healthcare services in England.

### Study design

We will use National Health Service (NHS) administrative data to characterise acute hospital admissions, outpatient attendances and emergency department (ED) attendances for primary MH disorders in England for the 5 years 2017 to 2021/22 (or the latest available data).

The data subjects for this project are all CYP aged 5–18 who have been admitted to hospital, attended A&E or had planned outpatient activity for any cause (physical or MH complaints) in England between 2017 and 2022 (or the latest available data).

We will purchase pseudo-anonymised individual-level data from the following data sets: HES admitted patient care (APC), HES outpatient care (OPC), HES A&E, HES Emergency Care Dataset (ECDS) and Mental Health Dataset (MHDS).

These data will allow the objectives described above to be achieved as follows:

Objective 1 of this project is to describe trends in healthcare activity related to MH problems in secondary care in England between 2017 and 2022 (or the latest available data). To do this, we will need to analyse secondary inpatient and outpatient MH care activity (held within MHDS and HES OPC), attendances to A&E due to MH concerns (data held within ECDS) and MH care activity within acute inpatient services (held within HES APC). We will describe trends over time, by age group, sex, markers of deprivation, prior MH healthcare activity and comorbid physical health problems and analyse geographic variation in MH healthcare activity within England.

In addition to sociodemographic characteristics of CYP attending secondary healthcare due to MH concerns, we will describe prior attendance to A&E (data held within HEE ECDS and HES A+E), prior admissions to acute paediatric services (data held within HES APC), prior outpatient MH activity (data held within MHDS and HES OPC) and prior MH inpatient activity (data held within MHDS).

We will identify healthcare activity (inpatient, outpatient, A&E) related to MH and physical disorders as follows:

1. Primary diagnostic ICD-10 codes (DIAG_1) relating to mental and physical health disorders and provider codes for treating consultant (TREATSPEF and MAINSPEF) within finished consultant episodes within HES APC.
2. Provider codes for treating consultant (TREATSPEF and MAINSPEF) for planned outpatient activity relating to mental and physical health specialty within HES OPC.
3. ECDS codes for MH presentations linked to a same-day acute admission with a primary diagnosis consistent with a mental or physical health presentation. This approach is only possible for 2018 onwards and cannot be done for pre-2018 ED data as reasons for presentation are not available.
4. Diagnostic coding within MHDS to identify MH outpatient activity not identified within HES OPC.

We will link, clean and collapse data using established protocols, we have developed for HES analyses. We will then describe:

1. Burden and trends in healthcare activity related to MH disorders by sex, ethnicity, level of deprivation and geographic region, and examine variation at Trust level where numbers allow.
2. Burden and trends in acuity of MH-related inpatient admissions. This will be examined by examining repeat admissions and readmissions and the numbers of admissions under the MH act.
3. Burden and trends in admission source and discharge destination of healthcare activity related to MH disorders (ie, MH ‘tier 4’ inpatients unit, criminal justice system, etc).
4. Burden and trends in healthcare activity related to MH disorders by MH diagnosis (note diagnostic coding within outpatients is limited). This will be limited to large groups, for example, eating disorder admissions, anxiety and depression, psychotic disorders—grouping to be determined.
5. Burden and trends in healthcare activity related to MH disorders associated with other medical conditions and comorbidities, identified through primary and secondary diagnostic coding within HES. We will particularly examine the common chronic conditions (diabetes, asthma, epilepsy) as well as use broader definitions of medical comorbidity.
6. Burden and trends in healthcare activity related to non-MH disorders within CYP identified as having healthcare activity related to MH disorders, including eating disorders, anxiety, depression and psychotic disorders—grouping used to define MH disorder to be determined.

Objective 2 of this study seeks to understand the financial impact of admissions to acute inpatient services due to MH problems. To do this, we need to first identify and group all hospitalisations in England in CYP due to MH concerns in acute inpatient services (objective 1). We will then use data within the National Cost Collection for NHS to estimate the financial burden associated with admitting CYP to an acute hospital inpatient unit for all causes and those related to MH admissions. We will then extrapolate this cost to a national estimate and describe changes in the financial burden of MH admission to acute inpatient paediatric services over time, and this varies by different parts of England.

Objective 3 of this study seeks to examine the role of primary and community healthcare services both before and after admission of CYP with MH conditions to and from paediatric wards. We will seek to use quality of community MH provision as a predictor for regional variation in the numbers of CYP admitted to acute inpatient units related to MH crises. We will do this by establishing the quality of community MH service provision using publicly available data, including staffing levels within general practice, and quality outcome framework indicators related to MH. We will also use data within the MHDS to assess community provision of MH services, including proxy indicators including wait time from referral to the first appointment. For this indicator, we require data held within MHDS.

### Patient

We presented our research proposal to members of Think4Brum, which is the youth advisory group for Forward Thinking Birmingham, and the GOSH Young Persons’ Advisory Group for research as part of a PPIE initiative. Focus groups of 40 young people (aged <18) and parents were held to discuss the acceptability of the methods and the use of HES data without consent. Feedback from these groups showed CYP believe this is an important area to research and the use of patient-identifiable data without consent is acceptable within this study.

### Data protection

This study is registered under reference No Z6364106/2023/03/96 health research in line with UCL’s Data Protection Policy.

NHS England data will be stored and analysed entirely within the UCL data safe haven (DSH), which has been certified to the ISO27001 information security standard and conforms to the NHS Information Governance Toolkit. Access to these data will be limited to those UCL employees contributing to this project. Data will be kept within the European Economic Area. The data will be encrypted for transfer, and information compliance training for information security, freedom of information and data protection will be completed by all staff who have access to the data. Data will be fully anonymised prior to the analysis and then extracted from UCL DSH after analysis.

The data will not be linked with any record-level data. There will be no requirement nor attempt to reidentify individuals from the data. The data will not be made available to any third parties other than those specified except in the form of aggregated outputs with small numbers suppressed in line with the HES analysis guide.

**Figure 2:**
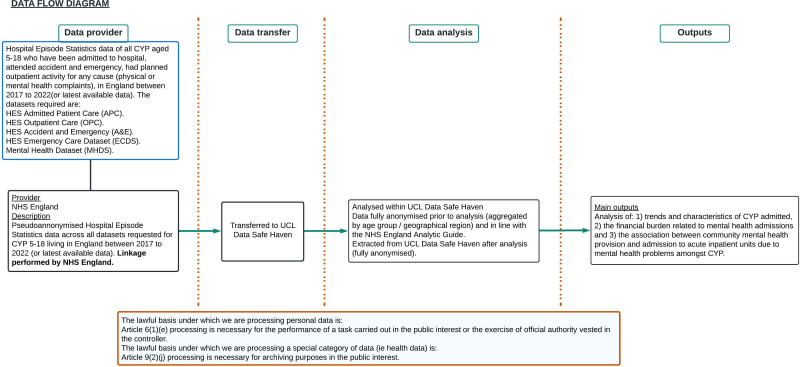
MAPS work package flow diagram. CAG, Confidentially Advisory Group; CYP, Children and Young People; ED, emergency department; HCP, Health Care Professionals; MAPS, Mental Health Admissions to Paediatric Wards Study; MH, mental health; ToC, theory of change; PPI, patient and public involvement.

A summary of our study procedure and lawful basis under which we are processing the data is seen in figure 2.

## Research ethics approval

This study was approved by Northwest—Preston Research Ethics Committee (Ref 23/NW/0192). We will not directly inform each data subject of the specific data transfer. Information regarding data transfer will be on our project’s website for the public to see (https://www.ucl.ac.uk/child-health/research/population-policy-and-practice-research-and-teaching-department/mental-health-admissions). We will be transparent with what data will be included and how it will be transferred, stored and analysed. Confidentiality advisory group approval was not required because the data requested are classified as pseudonymised data.

## Dissemination

See WP4 of the study in figure 1. As coproducers of the ToC, we will work with our stakeholder group to ensure wide dissemination of findings to effect change. Potential impacts will be on service development, new models of care, training and workforce planning.

## Supplementary Material

Reviewer comments

Author's manuscript
