# Supplementary material for: Mental Health Admissions to Paediatric Wards Study (MAPS): a protocol for the analysis of Hospital Episode Statistics (HES) data
Source: BMJ Paediatr Open. 2024 Jan 29;8(1):e002352. doi: 10.1136/bmjpo-2023-002352 (PMC11145899; doi:10.1136/bmjpo-2023-002352)
Supplement: Author's manuscript [file bmjpo-2023-002352.draft_revisions.pdf]

BMJ Paediatrics Open

BMJ Paediatrics Open is committed to open peer review. As part of this commitment we make the peer review history of every article we publish publicly available.

When an article is published we post the peer reviewers' comments and the authors' responses online. We also post the versions of the paper that were used during peer review. These are the versions that the peer review comments apply to.

The versions of the paper that follow are the versions that were submitted during the peer review process. They are not the versions of record or the final published versions. They should not be cited or distributed as the published version of this manuscript.

BMJ Paediatrics Open is an open access journal and the full, final, typeset and author-corrected version of record of the manuscript is available on our site with no access controls, subscription charges or pay-per-view fees (http://bmjpaedsopen.bmj.com).

If you have any questions on BMJ Paediatrics Open's open peer review process please email info.bmjpo@bmj.com

BMJ Paediatrics Open

Mental Health Admissions to Paediatric Wards Study (MAPS): a protocol for the analysis of Hospital Episode Statistics (HES) data to identify and characterise trends in mental health admissions in acute paediatric wards in England.

Journal:	*BMJ Paediatrics Open*
Manuscript ID	bmjpo-2023-002352
Article Type:	Protocol
Date Submitted by the Author:	23-Oct-2023
Complete List of Authors:	Hudson, Lee; Great Ormond Street Institute of Child Health,<br>Ward, Joseph ; University College London Great Ormond Street Institute of Child Health<br>Vázquez-Vázquez, Adriana; University College London Great Ormond Street Institute of Child Health, Population, Policy and Practice<br>Settle, Kate; University College London Great Ormond Street Institute of Child Health<br>Cornaglia , Francesca; Queen Mary University of London<br>Gibson , Faith ; University College London Great Ormond Street Institute of Child Health<br>Phillips , Kirsty ; University College London Great Ormond Street Institute of Child Health<br>Mathews , Gabrielle ; CYP Transformation Team, NHS England and NHS Improvement London, London, UK<br>Roberts , Helen ; UCL<br>Roland, Damian; University of Leicester, Health Sciences; University Hospitals of Leicester NHS Trust, Paediatric Emergency Medicine Leicester Academic (PEMLA) Group<br>Nicholls, Dasha; Imperial College London Department of Brain Sciences, Psychiatry<br>Elphinstone, Holly; University College London Great Ormond Street Institute of Child Health<br>Viner, Russell; UCL Institute of Child Health,
Keywords:	Child Psychiatry, Epidemiology, Psychology, Statistics, Adolescent Health

SCHOLARONE™
Manuscripts

I, the Submitting Author has the right to grant and does grant on behalf of all authors of the Work (as defined in the below author licence), an exclusive licence and/or a non-exclusive licence for contributions from authors who are: i) UK Crown employees; ii) where BMJ has agreed a CC-BY licence shall apply, and/or iii) in accordance with the terms applicable for US Federal Government officers or employees acting as part of their official duties; on a worldwide, perpetual, irrevocable, royalty-free basis to BMJ Publishing Group Ltd ("BMJ") its licensees and where the relevant Journal is co-owned by BMJ to the co-owners of the Journal, to publish the Work in this journal and any other BMJ products and to exploit all rights, as set out in our licence.

The Submitting Author accepts and understands that any supply made under these terms is made by BMJ to the Submitting Author unless you are acting as an employee on behalf of your employer or a postgraduate student of an affiliated institution which is paying any applicable article publishing charge ("APC") for Open Access articles. Where the Submitting Author wishes to make the Work available on an Open Access basis (and intends to pay the relevant APC), the terms of reuse of such Open Access shall be governed by a Creative Commons licence – details of these licences and which Creative Commons licence will apply to this Work are set out in our licence referred to above.

Other than as permitted in any relevant BMJ Author's Self Archiving Policies, I confirm this Work has not been accepted for publication elsewhere, is not being considered for publication elsewhere and does not duplicate material already published. I confirm all authors consent to publication of this Work and authorise the granting of this licence.

Confidential: For Review Only

Mental Health Admissions to Paediatric Wards Study (MAPS): a protocol for the analysis of Hospital Episode Statistics (HES) data to identify and characterise trends in mental health admissions in acute paediatric wards in England.

Lee Hudson[1,2*], Joseph Ward[1], Adriana Vázquez-Vázquez[1], Kate Settle[1], Francesca Cornaglia[3], Faith Gibson[1,2,4], Kirsty Phillips[1], Gabrielle Mathews[5], Helen Roberts[1], Damian Roland[6], Dasha Nicholls[7], Holly Elphinstone[1], Russell Viner[1]

1. Population, Policy and Practice Research Programme, UCL Great Ormond Street Institute of Child Health, London, UK
2. Great Ormond Street Hospital for Children NHS Trust, London, UK
3. Queen Mary University of London, London, UK
4. University of Surrey, Guildford, UK
5. CYP Transformation Team, NHS England and NHS Improvement London, London, UK
6. University Hospitals of Leicester NHS Trust, Leicester, UK
7. Division of Psychiatry, Imperial College London, London, UK

*Corresponding author:
Lee Hudson
Population, Policy and Practice Research Programme, UCL Great Ormond Street Institute of Child Health, London, UK
l.hudson@ucl.ac.uk

Confidential: For Review Only

ABSTRACT

Introduction

Children and young people (CYP) presenting with a mental health (MH) crisis are frequently admitted to general acute paediatric wards as a place of safety. Prior to the pandemic, a survey in England showed that CYP occupied 6% of general paediatric inpatient beds due to a MH crisis, and there have been longstanding concerns about the quality of care to support these patients in this setting. MAPS aims to generate a Theory of Change (ToC) model to improve the quality of care for CYP admitted to acute paediatric services after presenting in a MH crisis. Here we describe work package 1 (WP1) of the study, which has been granted ethics approval.

Methods and analysis

We will undertake a national (England), sequential, mixed methods study to inform a ToC framework alongside a stakeholder group consisting of patients, families/carers and health care professionals (HCPs). Our study consists of 4 work packages undertaken over 30 months. WP1 is limited to using national routine administrative data to identify and characterise trends in MH admissions in acute paediatric wards in England between 2015– 2022.

Ethics and dissemination

WP1 received ethical approval (Ref 23/NW/0192). We will publish the overall synthesis of data and the final ToC to improve care of CYP with MH crisis admitted to general acute paediatric settings. As co-producers of the ToC, we will work with our stakeholder group to ensure wide dissemination of findings. Potential impacts will be upon service development, new models of care, training and workforce planning.

What is already known on this topic

There is evidence that both the number of paediatric admissions and the severity of MH crisis in CYP have increased, and paediatric teams are experiencing challenges delivering safe care.

What this study hopes to add

- This study will provide a comprehensive national analysis describing trends and characteristics of acute admissions due to MH problems in CYP, and the financial burden associated with these,

in conjunction with an analysis of the association between community MH support and the local burden of acute admissions.
- This study will generate a ToC model to positively impact upon quality of care for CYP who are admitted to an acute paediatric service because of a MH crisis.

How this study might affect research, practice or policy

By producing a ToC approach, we expect to generate a system map to identify recommendations and transformation plans to share with policymakers, commissioners, service leads, and professionals.

INTRODUCTION

CYP presenting with a MH crisis are frequently admitted to general acute paediatric wards as a place of safety, despite not always having the resources or training (1, 2). Before the pandemic, a survey carried out in 2019 with 60% of acute paediatric services in England found that 6% of general paediatric beds were occupied by CYP with MH problems (3). Moreover, data from London suggest that the management of CYP with MH problems was one of the main challenges for acute children's services (3).

The rise in MH problems amongst CYP during the COVID-19 pandemic has been also well described (4,5). Recent national data report that being at high risk of MH problems rose from 1 in 9 in 2017 to 1 in 6 by 2021, with a doubling of the proportion of CYP at risk of eating problems over that same period (6). During the first wave, acute services became 'default providers' where community or inpatient CAMHS services were not accessible and during the third wave, admissions to acute wards appeared to peak (7). This mismatch of greater distress and reduced access led to increases in already unmet needs (8).

There is a striking lack of information available to guide care and service delivery for the rapidly growing issue of CYP with MH problems being admitted and managed on acute paediatric wards. In a review of top-line Hospital Episode Statistics (HES) data, MH admissions in children's wards increased around 25% over the 4 years prior to the pandemic (unpublished data, Viner), and anecdotal reports from within the paediatric community confirm that numbers of such admissions have increased.

Overall, MH admissions to acute paediatric wards are a long-standing issue that has been identified as a leading safety and quality concern for acute paediatric providers for some years (1,3). The MAPS study aims to generate a Theory of Change (ToC) model to improve the quality of care for CYP admitted to acute paediatric services after presenting in a MH crisis. Our study consists of 4 work packages. Here we describe work package 1 (WP1) which is limited to using national routine administrative data to identify and characterise trends in MH admissions in acute paediatric wards in England between 2015– 2022. WP2 and 3 were described separately (9).

INSERT FIGURE 1

METHODS AND ANALYSIS

We will use a ToC approach as our framework, which uses logic (quantitative) data and co-production (qualitative) data to map change. This approach has been applied to a range of areas of health and social care improvement settings (10-12).

We will undertake a national, sequential, mixed methods study to inform our ToC framework alongside a stakeholder group consisting of patients, families/carers and HCPs. Three work packages will deliver the types of evidence needed to inform our ToC. Here we describe the study design for WP1.

Confidential: For Review Only

Objectives

The analysis of routine administrative data in WP1 will be highly informative regarding national trends and characteristics of CYP admitted. The aims of WP1 are to:

1. Describe trends in MH admissions to acute inpatient services, attendances to accident & emergency (A&E), and outpatient appointments related to MH within England 2015- 2022 (or the latest available data) in CYP aged 5-18.
2. Understand the financial burden of MH admissions to acute paediatric wards among CYP aged 5-18 in England.
3. Examine the role of primary and community health care services in England.

Study design

We will use National Health Service (NHS) administrative data to characterise acute hospital admissions, outpatient attendances, and Emergency Department (ED) attendances for primary MH disorders in England for the 5 years 2017 to 2021/22 (or the latest available data).

The data subjects for this project are all CYP aged 5-18 who have been admitted to hospital, attended A&E, or had planned outpatient activity for any cause (physical or MH complaints) in England between 2017 to 2022 (or the latest available data).

We will purchase pseudo-anonymised individual level data from the following datasets: HES Admitted Patient Care (APC), HES Outpatient Care (OPC), HES Accident and Emergency (A&E), HES Emergency Care Dataset (ECDS) and Mental Health Dataset (MHDS).

These data will allow the objectives described above to be achieved as follows:

Objective 1 of this project is to describe trends in healthcare activity related to MH problems in secondary care in England between 2017-2022 (or the latest available data). To do this, we will need to analyse secondary inpatient and outpatient mental health care activity (held within MHDS and HES OPC), attendances to A&E due to MH concerns (data held within ECDS) and MH care activity within acute inpatient services (held within HES APC). We will describe trends over time, by age group, sex, markers of deprivation, prior MH healthcare activity and comorbid physical health problems, and analyse geographic variation in MH healthcare activity within England.

In addition to sociodemographic characteristics of CYP attending secondary healthcare due to MH concerns, we will describe prior attendance to A&E (data held within HEE ECDS and HES A+E), prior admissions to acute paediatric services (data held within HES APC), prior outpatient MH activity (data held within MHDS and HES OPC) and prior MH inpatient activity (data held within MHDS).

We will identify healthcare activity (inpatient, outpatient, A&E) related to MH and physical disorders as follows:

1. Primary diagnostic ICD-10 codes (DIAG_1) relating to mental and physical health disorders and provider codes for treating consultant (TREATSPEF and MAINSPEF) within finished consultant episodes within HES APC.
2. Provider codes for treating consultant (TREATSPEF and MAINSPEF) for planned outpatient activity relating to mental and physical health specialty within HES OPC.
3. ECDS codes for MH presentations linked to a same-day acute admission with a primary diagnosis consistent with a mental or physical health presentation. This approach is only possible for 2018 onwards and cannot be done for pre-2018 ED data as reasons for presentation are not available.

Confidential: For Review Only

4. Diagnostic coding within MHDS to identify MH outpatient activity not identified within HES OPC.

We will link, clean and collapse data using established protocols we have developed for HES analyses. We will then describe:

1. Burden and trends in healthcare activity related to MH disorders by sex, ethnicity, level of deprivation and geographic region, and examine variation at Trust level where numbers allow.
2. Burden and trends in acuity of mental health-related inpatient admissions. This will be examined by examining repeat admissions and readmissions and the numbers of admissions under the mental health act.
3. Burden and trends in admission source and discharge destination of healthcare activity related to MH disorders (i.e., MH "tier 4" inpatients unit, criminal justice system, etc).
4. Burden and trends in healthcare activity related to MH disorders by MH diagnosis (note diagnostic coding within outpatients is limited). This will be limited to large groups, e.g., eating disorder admissions, anxiety and depression, psychotic disorders – grouping to be determined.
5. Burden and trends in healthcare activity related to MH disorders associated with other medical conditions and comorbidities, identified through primary and secondary diagnostic coding within HES. We will particularly examine the common chronic conditions (diabetes, asthma, epilepsy) as well as use broader definitions of medical comorbidity.
6. Burden and trends in healthcare activity related to non-MH disorders within CYP identified as having healthcare activity related to MH disorders including eating disorders, anxiety, depression, and psychotic disorders – grouping used to define MH disorder to be determined.

Objective 2 of this project seeks to understand the financial impact of admissions to acute inpatient services due to MH problems. To do this, we need to first identify and group all hospitalisations in England in CYP due to MH concerns in acute inpatient services (objective 1). We will then use data within the National Cost Collection for NHS to estimate the financial burden associated with admitting CYP to an acute hospital inpatient unit for all causes, and those related to MH admissions. We will then extrapolate this cost to a national estimate and describe changes in the financial burden of MH admission to acute inpatient paediatric services over time, and this varies by different parts of England.

Objective 3 of this project seeks to examine the role of primary and community health care services both before and after admission of CYP with MH conditions to and from paediatric wards. We will seek to use quality of community MH provision as a predictor for regional variation in the numbers of CYP admitted to acute inpatient units related to MH crises. We will do this by establishing the quality of community MH service provision using publicly available data including staffing levels within general practice, and quality outcome framework indicators related to MH. We will also use data within the MHDS to assess community provision of MH services, including proxy indicators including wait time from referral to the first appointment. For this indicator, we require data held within MHDS.

<u>Patient & Public Involvement Statement</u>

We presented our research proposal to members of Think4Brum, which is the youth advisory group for Forward Thinking Birmingham, and the GOSH Young Persons' Advisory Group for research as part of a PPIE initiative. Focus groups of 40 young people (aged <18) and parents were held to discuss the acceptability of the methods and the use of HES data without consent. Feedback from these groups showed CYP believe this is an important area to research and the use of patient-identifiable data without consent is acceptable within this WP.

Confidential: For Review Only

Data protection

WP1 is registered under reference No Z6364106/2023/03/96 health research in line with UCL's Data Protection Policy.

NHS England data will be stored and analysed entirely within the UCL data safe haven (DSH), which has been certified to the ISO27001 information security standard and conforms to the NHS Information Governance Toolkit. Access to these data will be limited to those UCL employees contributing to this project. Data will be kept within the European Economic Area. The data will be encrypted for transfer, and information compliance training for information security, freedom of information, and data protection will be completed by all staff who have access to the data. Data will be fully anonymised prior to the analysis and then, extracted from UCL DSH after analysis.

The data will not be linked with any record level data. There will be no requirement nor attempt to reidentify individuals from the data. The data will not be made available to any third parties other than those specified except in the form of aggregated outputs with small numbers suppressed in line with the HES analysis guide.

A summary of our study procedure for WP1 and lawful basis under which we are processing the data can be seen in Figure 2.

INSERT FIGURE 2

RESEARCH ETHICS APPROVAL

WP1 was approved by Northwest - Preston Research Ethics Committee (Ref 23/NW/0192). We will not directly inform each data subject of the specific data transfer. Information regarding data transfer will be on our project's website for the public to see (https://www.ucl.ac.uk/child-health/research/population-policy-and-practice-research-and-teaching-department/mental-health-admissions). We will be transparent with what data will be included and how it will be transferred, stored and analysed. Confidentiality Advisory Group (CAG) approval was not required because the data requested are classified as pseudonymised data.

DISSEMINATION

See WP4 of the study in Figure 1. As co-producers of the ToC, we will work with our stakeholder group to ensure wide dissemination of findings to effect change. Potential impacts will be upon service development, new models of care, training and workforce planning.

REFERENCES

1. Royal College of Paediatrics and Child Health. Role of paediatricians in supporting children and young people's mental health – position statement. Report, 2020. https://www.rcpch.ac.uk/resources/role-paediatricians-supporting-children-youngpeoples-mental-health-position-statement
2. Review of acute care services for children and young people: Healthy London Partnership, London, 2017. https://www.healthylondon.org/resource/peer-reviewacute-care-services-children-young-people/
3. Royal College of Paediatrics and Child Health. A snapshot of general paediatric services and workforce in the UK. Report, 2020.
4. Ford T, John A, Gunnell D. Mental health of children and young people during pandemic. BMJ. 2021 Mar 10;372:n614. doi: 10.1136/bmj.n614. Erratum in: BMJ. 2021 Mar 19;372:n765. PMID: 33692087.

Confidential: For Review Only

5. Samji H, Wu J, Ladak A, Vossen C, Stewart E, Dove N, Long D, Snell G. Review: Mental health impacts of the COVID-19 pandemic on children and youth – a systematic review. Child Adolesc Ment Health. 2021 Aug 28. doi: 10.1111/camh.12501. Epub ahead of print. PMID: 34455683.
6. NHS Digitial. Mental Health of Children and Young People in England, 2021: Wave 2 follow up to the 2017 survey: NHS Digital, Health and Social Care Information Centre, 2021. https://files.digital.nhs.uk/97/B09EF8/mhcyp_2021_rep.pdf
7. Hudson, L. D., Chapman, S., Street, K. N., Nicholls, D., Roland, D., Dubicka, B., … Viner, R. M. (2022). Increased admissions to paediatric wards with a primary mental health diagnosis: results of a survey of a network of eating disorder paediatricians in England. Archives of Disease in Childhood, 107(3), 309 LP – 310. https://doi.org/10.1136/archdischild-2021-322700
8. Dubicka et al. (2017). Mental health services for children fail to meet soaring demand. BMJ 2017 Sep 29;358:j4254. Doi: 10.1136/bmj.j4254.
9. Hudson, L., Vázquez-Vázquez, A., Gibson, F., Phillips, K., Mathews, G., Roberts, H., … Viner, R. (in press). Mental Health Admissions to Paediatric Wards Study (MAPS): protocol of a prospective study of mental health admissions to paediatric wards in England using surveillance and qualitative methods. BMJ Paediatrics Open.
10. Wilkinson H, Hills D, Penn A, Barbrook-Johnson P (2021) Building a system-based Theory of Change using participatory systems mapping. Evaluation 27(1) 80-101.
11. Maini R, Mounier-Jack NS, Borghi J (2018) How to and how not to develop a theory of change to evaluate a complex intervention-reflections on an experience in the Democratic Republic of Congo BMJ Global Health 3: e000617.
12. Breuer E, Lee L, DeSilva M, Lund C (2016) Using theory of change to design and evaluate public health interventions-a systematic review Implementation Science 11:63.

AUTHOR'S CONTRIBUTIONS

RV, JW, FC and LH were major contributors to the development of the study protocol. AVV drafted the manuscript. All authors contributed to revising the manuscript and approved the final version.

FUNDING STATEMENT

This study is supported by the National Institute for Health and Care Research (NIHR) grant/award number 135036.

COMPETING INTEREST STATEMENT

None declared.

Figure 1. MAPS work package flow diagram
ED: Emergency department; MH: Mental Health; ToC: Theory of change; PPI: Patient and Public Involvement; CYP: Children and Young People; HCP: Health Care Professionals; CAG: Confidentially Advisory Group.

Figure 2. WP1 data flow diagram
HES: Hospital Episodes Statistics; CYP: Children and Young People; NHS: National Health Service; UCL: University College London; MH: Mental Health

Confidential: For Review Only

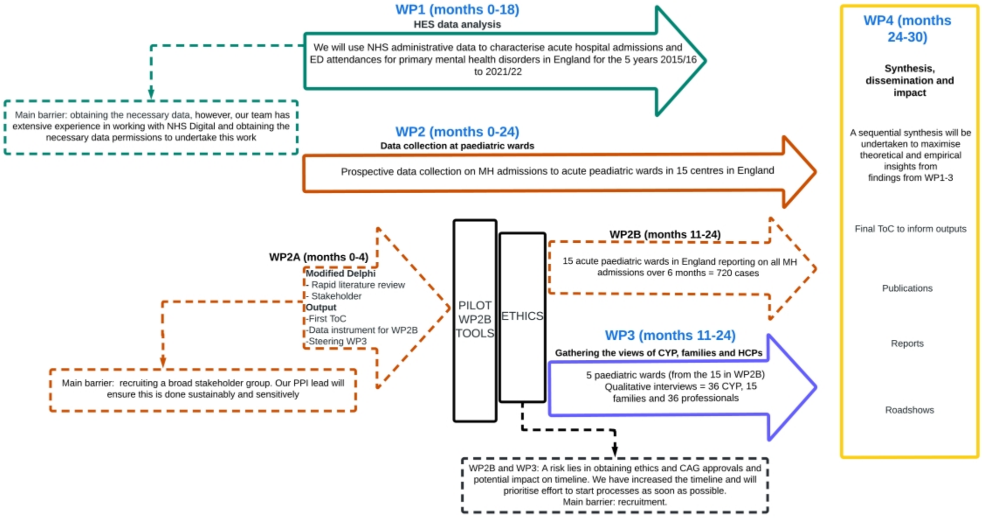

Figure 1. MAPS work package flow diagram

1243x670mm (118 x 118 DPI)

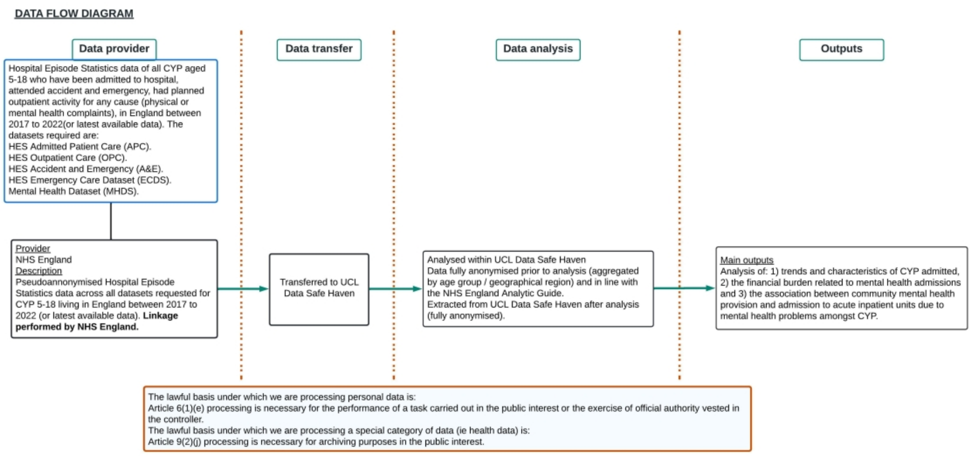

Figure 2. WP1 data flow diagram

454x216mm (300 x 300 DPI)

BMJ Paediatrics Open

Mental Health Admissions to Paediatric Wards Study (MAPS): a protocol for the analysis of Hospital Episode Statistics (HES) data.

Journal:	*BMJ Paediatrics Open*
Manuscript ID	bmjpo-2023-002352.R1
Article Type:	Protocol
Date Submitted by the Author:	20-Nov-2023

Complete List of Authors:	Hudson, Lee; Great Ormond Street Institute of Child Health,<br>Ward, Joseph ; University College London Great Ormond Street Institute of Child Health<br>Vázquez-Vázquez, Adriana; University College London Great Ormond Street Institute of Child Health, Population, Policy and Practice<br>Settle, Kate; University College London Great Ormond Street Institute of Child Health<br>Cornaglia , Francesca; Queen Mary University of London<br>Gibson , Faith ; University College London Great Ormond Street Institute of Child Health<br>Phillips , Kirsty ; University College London Great Ormond Street Institute of Child Health<br>Mathews , Gabrielle ; CYP Transformation Team, NHS England and NHS Improvement London, London, UK<br>Roberts , Helen ; UCL<br>Roland, Damian; University of Leicester, Health Sciences; University Hospitals of Leicester NHS Trust, Paediatric Emergency Medicine Leicester Academic (PEMLA) Group<br>Nicholls, Dasha; Imperial College London Department of Brain Sciences, Psychiatry<br>Elphinstone, Holly; University College London Great Ormond Street Institute of Child Health<br>Viner, Russell; UCL Institute of Child Health,

Keywords:	Child Psychiatry, Epidemiology, Psychology, Statistics, Adolescent Health

SCHOLARONE™
Manuscripts

I, the Submitting Author has the right to grant and does grant on behalf of all authors of the Work (as defined in the below author licence), an exclusive licence and/or a non-exclusive licence for contributions from authors who are: i) UK Crown employees; ii) where BMJ has agreed a CC-BY licence shall apply, and/or iii) in accordance with the terms applicable for US Federal Government officers or employees acting as part of their official duties; on a worldwide, perpetual, irrevocable, royalty-free basis to BMJ Publishing Group Ltd ("BMJ") its licensees and where the relevant Journal is co-owned by BMJ to the co-owners of the Journal, to publish the Work in this journal and any other BMJ products and to exploit all rights, as set out in our licence.

The Submitting Author accepts and understands that any supply made under these terms is made by BMJ to the Submitting Author unless you are acting as an employee on behalf of your employer or a postgraduate student of an affiliated institution which is paying any applicable article publishing charge ("APC") for Open Access articles. Where the Submitting Author wishes to make the Work available on an Open Access basis (and intends to pay the relevant APC), the terms of reuse of such Open Access shall be governed by a Creative Commons licence – details of these licences and which Creative Commons licence will apply to this Work are set out in our licence referred to above.

Other than as permitted in any relevant BMJ Author's Self Archiving Policies, I confirm this Work has not been accepted for publication elsewhere, is not being considered for publication elsewhere and does not duplicate material already published. I confirm all authors consent to publication of this Work and authorise the granting of this licence.

Confidential: For Review Only

Mental Health Admissions to Paediatric Wards Study (MAPS): a protocol for the analysis of Hospital Episode Statistics (HES) data.

Lee Duncan Hudson[1,2*], Joseph Ward[1], Adriana Vázquez-Vázquez[1], Kate Settle[1], Francesca Cornaglia[3], Faith Gibson[1,2,4], Kirsty Phillips[1], Gabrielle Mathews[5], Helen Roberts[1], Damian Roland[6], Dasha Nicholls[7], Holly Elphinstone[1], Russell Viner[1]

1. Population, Policy and Practice Research Programme, UCL Great Ormond Street Institute of Child Health, London, UK
2. Great Ormond Street Hospital for Children NHS Trust, London, UK
3. Queen Mary University of London, London, UK
4. University of Surrey, Guildford, UK
5. CYP Transformation Team, NHS England and NHS Improvement London, London, UK
6. University Hospitals of Leicester NHS Trust, Leicester, UK
7. Division of Psychiatry, Imperial College London, London, UK

*Corresponding author:
Lee Hudson
Population, Policy and Practice Research Programme, UCL Great Ormond Street Institute of Child Health, London, UK
l.hudson@ucl.ac.uk

ABSTRACT

Introduction

Children and young people (CYP) presenting with a mental health (MH) crisis are frequently admitted to general acute paediatric wards as a place of safety. Prior to the pandemic, a survey in England showed that CYP occupied 6% of general paediatric inpatient beds due to a MH crisis, and there have been longstanding concerns about the quality of care to support these patients in this setting. MAPS aims to generate a Theory of Change (ToC) model to improve the quality of care for CYP admitted to acute paediatric services after presenting in a MH crisis.

Methods and analysis

We will undertake a national (England), sequential, mixed methods study to inform a ToC framework alongside a stakeholder group consisting of patients, families/carers and health care professionals (HCPs). Our study consists of 4 work packages (WP) undertaken over 30 months. WP1 is limited to using national routine administrative data to identify and characterise trends in MH admissions in acute paediatric wards in England between 2015– 2022.

Ethics and dissemination

WP1 received ethical approval (Ref 23/NW/0192). We will publish the overall synthesis of data and the final ToC to improve care of CYP with MH crisis admitted to general acute paediatric settings. As co-producers of the ToC, we will work with our stakeholder group to ensure wide dissemination of findings. Potential impacts will be upon service development, new models of care, training and workforce planning.

What is already known on this topic

There is evidence that both the number of paediatric admissions and the severity of MH crisis in CYP have increased, and paediatric teams are experiencing challenges delivering safe care.

What this study hopes to add

- This study will provide a comprehensive national analysis describing trends and characteristics of acute admissions due to MH problems in CYP, and the financial burden associated with these, in conjunction with an analysis of the association between community MH support and the local burden of acute admissions.

Confidential: For Review Only

- This study will generate a ToC model to positively impact upon quality of care for CYP who are admitted to an acute paediatric service because of a MH crisis.

How this study might affect research, practice or policy

By producing a ToC approach, we expect to generate a system map to identify recommendations and transformation plans to share with policymakers, commissioners, service leads, and professionals.

INTRODUCTION

CYP presenting with a MH crisis are frequently admitted to general acute paediatric wards as a place of safety, despite not always having the resources or training (1, 2). Before the pandemic, a survey carried out in 2019 with 60% of acute paediatric services in England found that 6% of general paediatric beds were occupied by CYP with MH problems (3). Moreover, data from London suggest that the management of CYP with MH problems was one of the main challenges for acute children's services (3).

The rise in MH problems amongst CYP during the COVID-19 pandemic has been also well described (4,5). Recent national data report that being at high risk of MH problems rose from 1 in 9 in 2017 to 1 in 6 by 2021, with a doubling of the proportion of CYP at risk of eating problems over that same period (6). During the first wave, acute services became 'default providers' where community or inpatient CAMHS services were not accessible and during the third wave, admissions to acute wards appeared to peak (7). This mismatch of greater distress and reduced access led to increases in already unmet needs (8).

There is a striking lack of information available to guide care and service delivery for the rapidly growing issue of CYP with MH problems being admitted and managed on acute paediatric wards. Evidence on interventions to avoid inpatient admissions for CYP presenting in MH crisis is poor and limited (9), meaning that CYP are likely to continue to need to be admitted in crisis, with paediatric wards a commonplace whilst awaiting assessment given the lack of direct access to a specialist MH ward form most CYP.

Overall, MH admissions to acute paediatric wards are a long-standing issue that has been identified as a leading safety and quality concern for acute paediatric providers for some years (1,3). The MAPS study aims to generate a Theory of Change (ToC) model to improve the quality of care for CYP admitted to acute paediatric services after presenting in a MH crisis. Our study consists of 4 work packages. Here we describe work package 1 (WP1) which is limited to using national routine administrative data to identify and characterise trends in MH admissions in acute paediatric wards in England between 2015– 2022. WP2 and 3 were described separately (10).

INSERT FIGURE 1

METHODS AND ANALYSIS

We will use a ToC approach as our framework, which uses logic (quantitative) data and co-production (qualitative) data to map change. This approach has been applied to a range of areas of health and social care improvement settings (11-13).

We will undertake a national, sequential, mixed methods study to inform our ToC framework alongside a stakeholder group consisting of patients, families/carers and HCPs. Three work packages will deliver the types of evidence needed to inform our ToC. Here we describe the study design for WP1.

Confidential: For Review Only

Objectives

The analysis of routine administrative data will be highly informative regarding national trends and characteristics of CYP admitted. The aims are to:

1. Describe trends in MH admissions to acute inpatient services, attendances to accident & emergency (A&E), and outpatient appointments related to MH within England 2015- 2022 (or the latest available data) in CYP aged 5-18.
2. Understand the financial burden of MH admissions to acute paediatric wards among CYP aged 5-18 in England.
3. Examine the role of primary and community health care services in England.

Study design

We will use National Health Service (NHS) administrative data to characterise acute hospital admissions, outpatient attendances, and Emergency Department (ED) attendances for primary MH disorders in England for the 5 years 2017 to 2021/22 (or the latest available data).

The data subjects for this project are all CYP aged 5-18 who have been admitted to hospital, attended A&E, or had planned outpatient activity for any cause (physical or MH complaints) in England between 2017 to 2022 (or the latest available data).

We will purchase pseudo-anonymised individual level data from the following datasets: HES Admitted Patient Care (APC), HES Outpatient Care (OPC), HES Accident and Emergency (A&E), HES Emergency Care Dataset (ECDS) and Mental Health Dataset (MHDS).

These data will allow the objectives described above to be achieved as follows:

Objective 1 of this project is to describe trends in healthcare activity related to MH problems in secondary care in England between 2017-2022 (or the latest available data). To do this, we will need to analyse secondary inpatient and outpatient mental health care activity (held within MHDS and HES OPC), attendances to A&E due to MH concerns (data held within ECDS) and MH care activity within acute inpatient services (held within HES APC). We will describe trends over time, by age group, sex, markers of deprivation, prior MH healthcare activity and comorbid physical health problems, and analyse geographic variation in MH healthcare activity within England.

In addition to sociodemographic characteristics of CYP attending secondary healthcare due to MH concerns, we will describe prior attendance to A&E (data held within HEE ECDS and HES A+E), prior admissions to acute paediatric services (data held within HES APC), prior outpatient MH activity (data held within MHDS and HES OPC) and prior MH inpatient activity (data held within MHDS).

We will identify healthcare activity (inpatient, outpatient, A&E) related to MH and physical disorders as follows:

1. Primary diagnostic ICD-10 codes (DIAG_1) relating to mental and physical health disorders and provider codes for treating consultant (TREATSPEF and MAINSPEF) within finished consultant episodes within HES APC.
2. Provider codes for treating consultant (TREATSPEF and MAINSPEF) for planned outpatient activity relating to mental and physical health specialty within HES OPC.
3. ECDS codes for MH presentations linked to a same-day acute admission with a primary diagnosis consistent with a mental or physical health presentation. This approach is only possible for 2018 onwards and cannot be done for pre-2018 ED data as reasons for presentation are not available.

Confidential: For Review Only

4. Diagnostic coding within MHDS to identify MH outpatient activity not identified within HES OPC.

We will link, clean and collapse data using established protocols we have developed for HES analyses. We will then describe:

1. Burden and trends in healthcare activity related to MH disorders by sex, ethnicity, level of deprivation and geographic region, and examine variation at Trust level where numbers allow.
2. Burden and trends in acuity of mental health-related inpatient admissions. This will be examined by examining repeat admissions and readmissions and the numbers of admissions under the mental health act.
3. Burden and trends in admission source and discharge destination of healthcare activity related to MH disorders (i.e., MH “tier 4” inpatients unit, criminal justice system, etc).
4. Burden and trends in healthcare activity related to MH disorders by MH diagnosis (note diagnostic coding within outpatients is limited). This will be limited to large groups, e.g., eating disorder admissions, anxiety and depression, psychotic disorders – grouping to be determined.
5. Burden and trends in healthcare activity related to MH disorders associated with other medical conditions and comorbidities, identified through primary and secondary diagnostic coding within HES. We will particularly examine the common chronic conditions (diabetes, asthma, epilepsy) as well as use broader definitions of medical comorbidity.
6. Burden and trends in healthcare activity related to non-MH disorders within CYP identified as having healthcare activity related to MH disorders including eating disorders, anxiety, depression, and psychotic disorders – grouping used to define MH disorder to be determined.

Objective 2 of this study seeks to understand the financial impact of admissions to acute inpatient services due to MH problems. To do this, we need to first identify and group all hospitalisations in England in CYP due to MH concerns in acute inpatient services (objective 1). We will then use data within the National Cost Collection for NHS to estimate the financial burden associated with admitting CYP to an acute hospital inpatient unit for all causes, and those related to MH admissions. We will then extrapolate this cost to a national estimate and describe changes in the financial burden of MH admission to acute inpatient paediatric services over time, and this varies by different parts of England.

Objective 3 of this study seeks to examine the role of primary and community health care services both before and after admission of CYP with MH conditions to and from paediatric wards. We will seek to use quality of community MH provision as a predictor for regional variation in the numbers of CYP admitted to acute inpatient units related to MH crises. We will do this by establishing the quality of community MH service provision using publicly available data including staffing levels within general practice, and quality outcome framework indicators related to MH. We will also use data within the MHDS to assess community provision of MH services, including proxy indicators including wait time from referral to the first appointment. For this indicator, we require data held within MHDS.

Patient & Public Involvement Statement

We presented our research proposal to members of Think4Brum, which is the youth advisory group for Forward Thinking Birmingham, and the GOSH Young Persons’ Advisory Group for research as part of a PPIE initiative. Focus groups of 40 young people (aged <18) and parents were held to discuss the acceptability of the methods and the use of HES data without consent. Feedback from these groups showed CYP believe this is an important area to research and the use of patient-identifiable data without consent is acceptable within this study.

Confidential: For Review Only

Confidential: For Review Only

Data protection

This study is registered under reference No Z6364106/2023/03/96 health research in line with UCL's Data Protection Policy.

NHS England data will be stored and analysed entirely within the UCL data safe haven (DSH), which has been certified to the ISO27001 information security standard and conforms to the NHS Information Governance Toolkit. Access to these data will be limited to those UCL employees contributing to this project. Data will be kept within the European Economic Area. The data will be encrypted for transfer, and information compliance training for information security, freedom of information, and data protection will be completed by all staff who have access to the data. Data will be fully anonymised prior to the analysis and then, extracted from UCL DSH after analysis.

The data will not be linked with any record level data. There will be no requirement nor attempt to reidentify individuals from the data. The data will not be made available to any third parties other than those specified except in the form of aggregated outputs with small numbers suppressed in line with the HES analysis guide.

A summary of our study procedure and lawful basis under which we are processing the data can be seen in Figure 2.

INSERT FIGURE 2

RESEARCH ETHICS APPROVAL

This study was approved by Northwest - Preston Research Ethics Committee (Ref 23/NW/0192). We will not directly inform each data subject of the specific data transfer. Information regarding data transfer will be on our project's website for the public to see (https://www.ucl.ac.uk/child-health/research/population-policy-and-practice-research-and-teaching-department/mental-health-admissions). We will be transparent with what data will be included and how it will be transferred, stored and analysed. Confidentiality Advisory Group (CAG) approval was not required because the data requested are classified as pseudonymised data.

DISSEMINATION

See WP4 of the study in Figure 1. As co-producers of the ToC, we will work with our stakeholder group to ensure wide dissemination of findings to effect change. Potential impacts will be upon service development, new models of care, training and workforce planning.

REFERENCES

1. Royal College of Paediatrics and Child Health. Role of paediatricians in supporting children and young people's mental health – position statement. Report, 2020. https://www.rcpch.ac.uk/resources/role-paediatricians-supporting-children-youngpeoples-mental-health-position-statement
2. Review of acute care services for children and young people: Healthy London Partnership, London, 2017. https://www.healthylondon.org/resource/peer-reviewacute-care-services-children-young-people/
3. Royal College of Paediatrics and Child Health. A snapshot of general paediatric services and workforce in the UK. Report, 2020.
4. Ford T, John A, Gunnell D. Mental health of children and young people during pandemic. BMJ. 2021 Mar 10;372:n614. doi: 10.1136/bmj.n614. Erratum in: BMJ. 2021 Mar 19;372:n765. PMID: 33692087.

5. Samji H, Wu J, Ladak A, Vossen C, Stewart E, Dove N, Long D, Snell G. Review: Mental health impacts of the COVID-19 pandemic on children and youth – a systematic review. Child Adolesc Ment Health. 2021 Aug 28. doi: 10.1111/camh.12501. Epub ahead of print. PMID: 34455683.
6. NHS Digitial. Mental Health of Children and Young People in England, 2021: Wave 2 follow up to the 2017 survey: NHS Digital, Health and Social Care Information Centre, 2021. https://files.digital.nhs.uk/97/B09EF8/mhcyp_2021_rep.pdf
7. Hudson, L. D., Chapman, S., Street, K. N., Nicholls, D., Roland, D., Dubicka, B., … Viner, R. M. (2022). Increased admissions to paediatric wards with a primary mental health diagnosis: results of a survey of a network of eating disorder paediatricians in England. Archives of Disease in Childhood, 107(3), 309 LP – 310. https://doi.org/10.1136/archdischild-2021-322700
8. Dubicka et al. (2017). Mental health services for children fail to meet soaring demand. BMJ 2017 Sep 29;358:j4254. Doi: 10.1136/bmj.j4254.
9. Clisu, D. A., Layther, I., Dover, D., Viner, R. M., Read, T., Cheesman, D., ... & Hudson, L. D. (2022). Alternatives to mental health admissions for children and adolescents experiencing mental health crises: A systematic review of the literature. Clinical child psychology and psychiatry, 27(1), 35-60.
10. Hudson, L., Vázquez-Vázquez, A., Gibson, F., Phillips, K., Mathews, G., Roberts, H., … Viner, R. (in press). Mental Health Admissions to Paediatric Wards Study (MAPS): protocol of a prospective study of mental health admissions to paediatric wards in England using surveillance and qualitative methods. BMJ Paediatrics Open.
11. Wilkinson H, Hills D, Penn A, Barbrook-Johnson P (2021) Building a system-based Theory of Change using participatory systems mapping. Evaluation 27(1) 80-101.
12. Maini R, Mounier-Jack NS, Borghi J (2018) How to and how not to develop a theory of change to evaluate a complex intervention-reflections on an experience in the Democratic Republic of Congo BMJ Global Health 3: e000617.
13. Breuer E, Lee L, DeSilva M, Lund C (2016) Using theory of change to design and evaluate public health interventions-a systematic review Implementation Science 11:63.

AUTHOR’S CONTRIBUTIONS

RV, JW, FC and LH were major contributors to the development of the study protocol. AVV drafted the manuscript. All authors contributed to revising the manuscript and approved the final version.

FUNDING STATEMENT

This study is supported by the National Institute for Health and Care Research (NIHR) grant/award number 135036.

COMPETING INTEREST STATEMENT

None declared.

Figure 1. MAPS work package flow diagram

ED: Emergency department; MH: Mental Health; ToC: Theory of change; PPI: Patient and Public Involvement; CYP: Children and Young People; HCP: Health Care Professionals; CAG: Confidentially Advisory Group.

Figure 2. WP1 data flow diagram

HES: Hospital Episodes Statistics; CYP: Children and Young People; NHS: National Health Service; UCL: University College London; MH: Mental Health

Confidential: For Review Only

Confidential: For Review Only

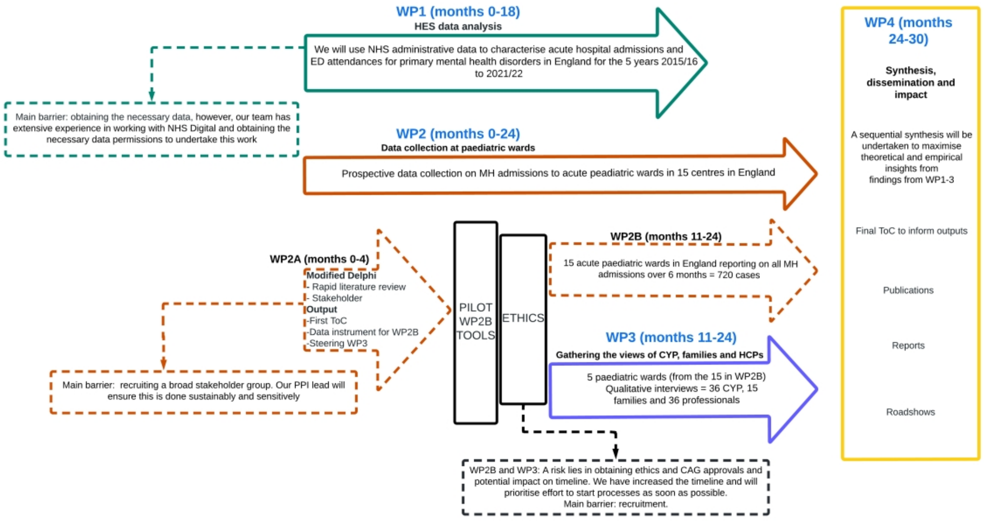

Figure 1. MAPS work package flow diagram

1243x670mm (118 x 118 DPI)

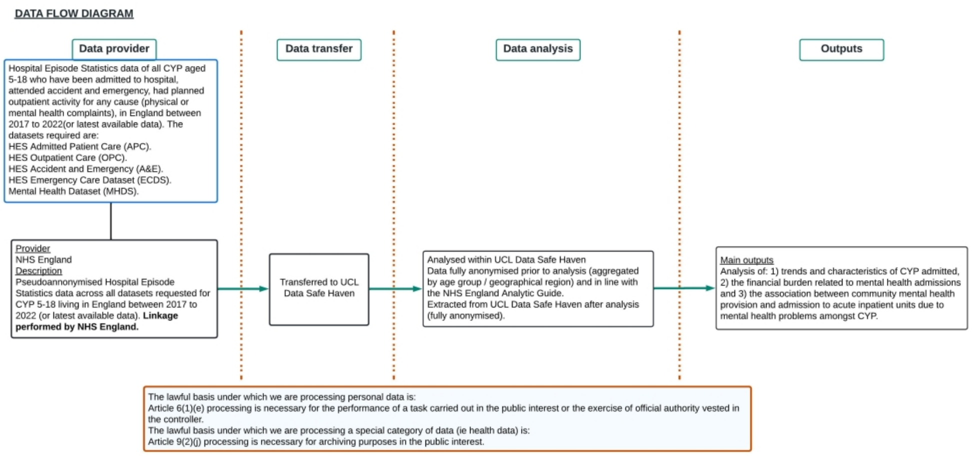

Figure 2. WP1 data flow diagram

454x216mm (300 x 300 DPI)
