# Supplementary material for: Mental Health Admissions to Paediatric Wards Study (MAPS): a protocol for the analysis of Hospital Episode Statistics (HES) data
Source: BMJ Paediatr Open. 2024 Jan 29;8(1):e002352. doi: 10.1136/bmjpo-2023-002352 (PMC11145899; doi:10.1136/bmjpo-2023-002352)
Supplement: Reviewer comments [file bmjpo-2023-002352.reviewer_comments.pdf]

PEER REVIEW HISTORY

BMJ Paediatrics Open publishes all reviews undertaken for accepted manuscripts. Reviewers are asked to complete a checklist review form and are provided with free text boxes to elaborate on their assessment. These free text comments are reproduced below.

ARTICLE DETAILS

**TITLE (PROVISIONAL)**	Mental Health Admissions to Paediatric Wards Study (MAPS): a protocol for the analysis of Hospital Episode Statistics (HES) data.
**AUTHORS**	Hudson, Lee Duncan<br>Ward, Joseph<br>Vázquez-Vázquez, Adriana<br>Settle, Kate<br>Cornaglia , Francesca<br>Gibson , Faith<br>Phillips , Kirsty<br>Mathews , Gabrielle<br>Roberts , Helen<br>Roland, Damian<br>Nicholls, Dasha E<br>Elphinstone, Holly<br>Viner, Russell

This article was not externally reviewed at BMJ Paediatrics Open. Protocols that have been independently assessed prior to submission to BMJ Paediatrics Open are usually fast-tracked to publication on the grounds that further substantial changes will not be possible. This independent assessment will usually be external, independent review for both a substantial grant award from a non-commercial or government funder and ethics approval.
